# Sirtuin 3 (SIRT3) maintains bone homeostasis by regulating AMPK-PGC-1β axis in mice

**DOI:** 10.1038/srep22511

**Published:** 2016-03-01

**Authors:** Jeong-Eun Huh, Ji Hye Shin, Eun Sun Jang, So Jeong Park, Doo Ri Park, Ryeojin Ko, Dong-Hyun Seo, Han-Sung Kim, Seoung Hoon Lee, Yongwon Choi, Hyun Seok Kim, Soo Young Lee

**Affiliations:** 1 Department of Life Science, Ewha Womans University, Seoul 120-750, Korea; 2 The Research Center for Cellular Homeostasis, Ewha Womans University, Seoul 120-750, Korea; 3 Department of Biomedical Engineering, College of Health Science, Institute of Medical Engineering, Yonsei University, Wonju 220-710, Korea; 4 Department of Oral Microbiology and Immunology, College of Dentistry, Wonkwang University, Iksan 570-749, Korea; 5 Department of Pathology and Laboratory Medicine, University of Pennsylvania Perelman School of Medicine, Philadelphia, PA 19104, USA; 6 Department of Bioinspired Science, Ewha Womans University, Seoul 120-750, Korea

## Abstract

The mitochondrial sirtuin 3 (SIRT3) is involved in suppressing the onset of multiple pathologies, including cardiovascular disease, fatty liver, age-related hearing loss, and breast cancer. But a physiological role of SIRT3 in bone metabolism is not known. Here we show that SIRT3 is a key regulatory molecule to maintain bone homeostasis. Mice deficient in SIRT3 exhibited severe osteopenia owing to increased numbers of osteoclasts. Osteoclast precursors from *Sirt3*−/− mice underwent increased osteoclastogenesis in response to receptor activator of nuclear factor-κB ligand (RANKL), an essential cytokine for osteoclast differentiation. SIRT3 expression from RANKL induction depended on the transcription coactivator PGC-1β (peroxisome proliferator-activated receptor-γ co-activator-1β) and the nuclear receptor ERRα (estrogen receptor-related receptor α), and that SIRT3 inhibited the differentiation by interfering with the RANKL-induced expression of PGC-1β. Thus an auto-regulatory feedback mechanism operates to induce its own inhibitor SIRT3 by PGC-1β. Moreover, *Sirt3*−/− osteoclast precursors reduced AMP-activated protein kinase (AMPK) phosphorylation through down-regulating the expression of AMPK. Our results suggest that a mitochondrial SIRT3 is an intrinsic inhibitor for RANKL-mediated osteoclastogenesis.

Bone remodeling depends on a delicate balance between bone resorption and bone formation, wherein bone-resorbing osteoclasts and bone-forming osteoblasts play essential roles^1^. Tipping this balance in favor of osteoclasts leads to pathological bone resorption, as seen in bone loss diseases such as rheumatoid arthritis and postmenopausal osteoporosis^1^,^2^. Thus, a tight regulation of osteoclast formation is essential for maintenance of bone homeostasis. Osteoclasts are derived from the monocyte-macrophages lineage. Upon binding of RANKL (receptor activator of nuclear factor κB ligand) to its receptor RANK at the surface of the osteoclast precursors, these cells undergo differentiation into mature osteoclasts that are multinucleated bone-resorbing cells^3^,^4^. Numerous studies have focused on the RANKL-induced genes, such as *Nfatc1*, c-*fos*, *Oscar*, *Ctsk* and *Calcr*, which encode NFATc1, c-Fos, osteoclast-associated receptor (OSCAR), cathepsin K and calcitonin receptor, respectively^5^,^6^,^7^,^8^, and their positive roles in osteoclast formation and function. In addition, a negative regulator induced by RANKL stimulation has been reported^9^. A typical example is interferon-β (IFN-β), which is secreted and binds its receptor, on neighboring osteoclast precursors, thereby inhibiting osteoclast differentiation. However, an intrinsic factor induced by RANKL that mediates a negative feedback mechanism to maintain bone homeostasis has not been fully elucidated.

Sirtuins, which are the mammalian homologues of the Sir2α in yeast, are the family of nicotinamide adenine dinucleotide (NAD)^+^-dependent protein deacetylases that regulate a myriad of cellular events including growth, apoptosis, DNA repair, cellular metabolisms, autophagy, etc., and are indeed involved with a variety of organismal physiology including inflammation, metabolisms, cancer, neurodegeneration, and aging^10^,^11^,^12^. Among the sirtuin family, SIRT3-5 are localized in mitochondria and affect the activity of multiple metabolic proteins of mitochondria^13^. In particular, SIRT3 deacetylates many proteins and regulates their activities, which are involved with mitochondrial biogenesis, ROS homeostasis, and metabolic pathways in mitochondria^14^,^15^,^16^. Previous studies have reported that SIRT3 is involved in the control of the mitochondrial ATP-production machinery through effects on the respiratory chain^17^,^18^, indicating that SIRT3 may be a critical mediator of energy required under a variety of stress conditions. In fact, SIRT3 may regulate the synthesis of ATP, at least in the heart and muscle, through regulation of AMP-activated protein kinase (AMPK), which acts as a sensor of cellular energy status^19^,^20^. Once activated, AMPK activates catabolic pathways, mostly by enhancing oxidative metabolism and mitochondrial biogenesis to produce ATP, while anabolic pathways that involve the expenditure of ATP are suppressed^21^. Although it is reasonable to expect that for the mechanical network between SIRT3 and AMPK depending on energy availability, the precise mechanism has not yet been covered. Moreover, the physiological roles of SIRT3 in several organs in mammal have been extensively documented with respect to mitochondrial metabolism^22^,^23^,^24^. However, it is still unknown whether SIRT3 is involved in bone homeostasis.

In this study, we revealed that *Sirt3*−/− osteoclast precursor cells had increased levels of osteoclast differentiation, and indeed found that *Sirt3*-deficient mice exhibit decreased bone mass due to an increased number of osteoclasts. SIRT3 was induced by PGC-1β together with ERRα during RANKL-induced osteoclast differentiation, and it may function as a negative mediator for osteoclastogenesis through stabilization of AMPK protein. Thus, our data suggest that SIRT3 plays an important role as a molecular brake on excessive osteoclastogenesis.

## Results

### SIRT3 null background mice had decreased bone mass

**Figure 1:**
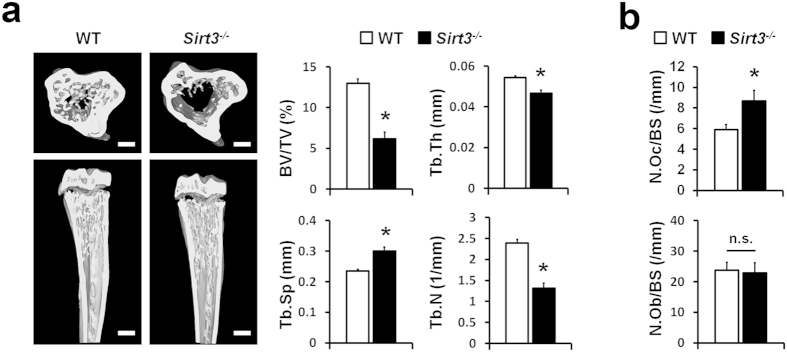
SIRT3 deletion decreased bone mass *in vivo*. (**a**) Microcomputed tomography (μCT) images of the tibias of 8-week-old wild type (WT) and *Sirt3*^*−/−*^ mice (*Left*), and quantification of trabecular bone volume and architecture (*Right*). BV/TV, bone volume per tissue volume; Tb.Th, trabecular thickness; Tb.Sp, trabecular spacing; Tb.N, trabecular number. *n* = 5 per group. *P < 0.001. Scale bar, 1 mm. (**b**) Quantification of osteoclasts and osteoblasts form histological analysis of tibias of WT and *Sirt3*^*−/−*^ mice. N.Oc/BS, osteoclast number per bone surface; N.Ob/BS, osteoblast number per bone surface. *n* = 6 per group. *P < 0.05. Data represent mean ± SD.

Since SIRT3 is known to promote mitochondrial biogenesis^14^,^15^, which plays a critical role in osteoclast formation^25^,^26^, we investigated the role of SIRT3 in osteoclasts *in vivo*. Microcomputed tomography (μCT) analysis clearly revealed that the bone volume fraction (BV/TV) was significantly reduced in mice deficient in *Sirt3* (*Sirt3*−/− mice). In addition, the trabecular thickness (Tb.Th) and trabecular number (Tb.N) were decreased in the *Sirt3*−/− bones, whereas trabecular separation (Tb.Sp) was increased (Fig. 1a). Bone morphometric analysis indicated that *Sirt3*−/− mice had significantly a higher number of TRAP-positive osteoclasts (N.Oc/BS), with no significant differences in osteoblast number on the surface of trabecular bones (N.Ob/BS) (Fig. 1b). In addition, the osteogenic potential of bone marrow-derived mesenchymal stem cells was indistinguishable in the wild type (WT) and *Sirt3*−/− mice (Supplementary Fig. 1). These results suggest that the abnormal bone phenotype in the *Sirt3*−/− mice was mainly caused by increased osteoclastogenesis *in vivo*.

### SIRT3 negatively regulated osteoclast differentiation

**Figure 2:**
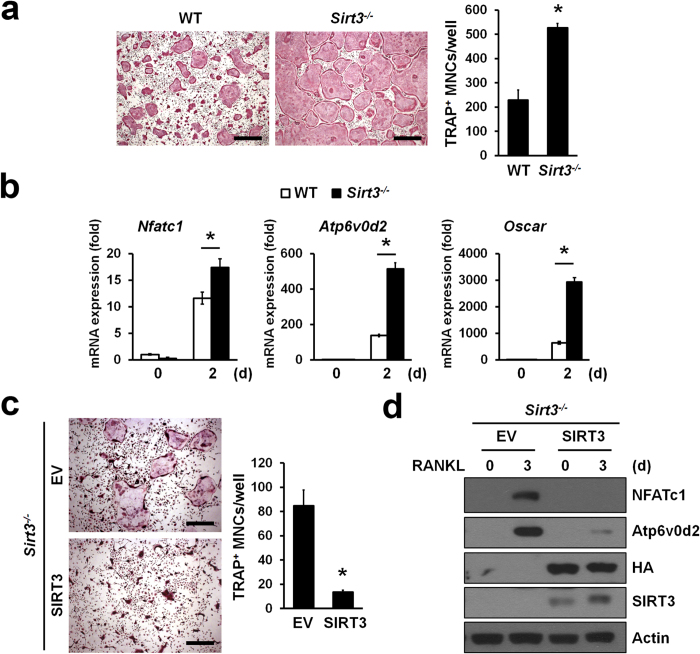
SIRT3 deficiency enhanced osteoclastogenesis. (**a**) BMMs from WT or *Sirt3*^*−/−*^ mice were stimulated with RANKL for 3 days. Cells were stained with TRAP solution. TRAP^+^ MNCs (>5 nuclei) were counted. Scale bar, 500 μm. *P < 0.01. Data are represented as mean ± SD. (**b**) BMMs from WT or *Sirt3*^*−/−*^ mice were stimulated with RANKL for 2 days. The mRNA expression of representative *Nfatc1* and NFATc1-induced genes *Atp6v0d2* and *Oscar* during osteoclast differentiation were determined by qRT-PCR. *P < 0.01 between the indicated groups. Data are represented as mean ± SD. (**c**) Osteoclast differentiation rescued by retroviruses expressing SIRT3 or empty vector (EV) in *Sirt3*^*−/−*^ BMMs. TRAP^+^MNCs (>5 nuclei) were counted. Scale bar, 500 μm. *P < 0.01. Data are represented as mean ± SD. (**d**) BMMs from *Sirt3*^*−/−*^ mice were transduced with retroviruses expressing HA-tagged SIRT3 or EV and were treated with RANKL for 3 days. NFATc1 and Atp6v0d2 protein expression was determined by immunoblot analysis. Actin serves as a loading control.

To investigate the intrinsic cellular role of SIRT3 in RANKL-induced osteoclastogenesis, we isolated bone marrow-derived monocytes/macrophages (BMMs) from *Sirt3*−/− mice and WT littermates and cultured them *ex vivo* in the presence of M-CSF and RANKL. *Sirt3*−/− BMMs exhibited significant increased numbers of TRAP-positive multinucleated cells (MNCs) in comparison to WT BMMs (Fig. 2a). Consistent with this, SIRT3 deletion resulted in a significantly increased level of *Nfatc1* mRNA, a key osteoclastogenic transcription factor, that of *Atp6v0d2* and *Oscar* that are known as osteoclast-specific genes (Fig. 2b), and also that of PGC-1β and its target genes that are induced during osteoclastogenesis (Supplementary Fig. 2a,b). The enhanced effects on osteoclastogenesis were completely rescued by retrovirus-mediated reintroduction of SIRT3 but not for control (EV, Empty Vector) into *Sirt3*−/− BMMs (Fig. 2c). In addition, reconstitution of SIRT3 in *Sirt3*−/− BMMs led to a reduction of NFATc1 and Atp6v0d2 protein levels (Fig. 2d). As such, SIRT3 is likely to function as a negative regulator of osteoclast differentiation.

**Figure 3:**
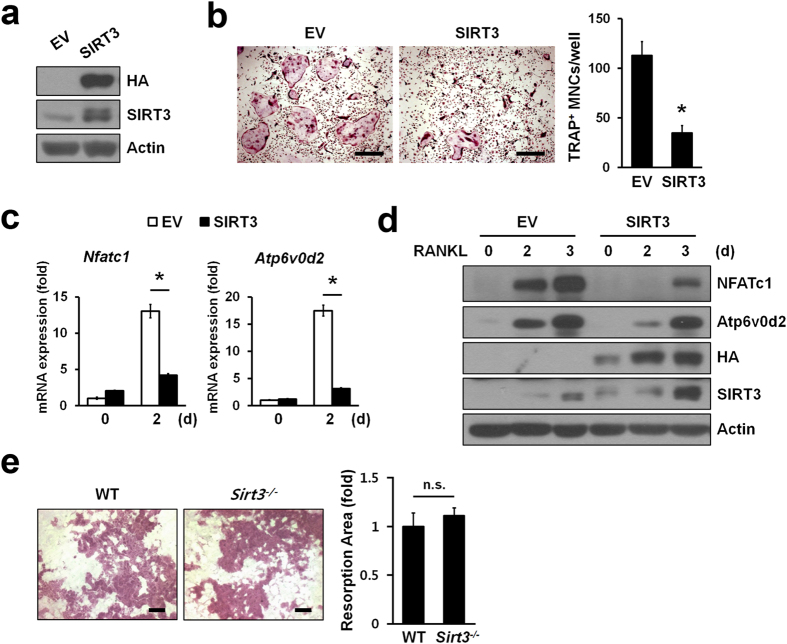
SIRT3 negatively regulated osteoclast differentiation but not function. (**a**) BMMs were infected with retroviruses expressing HA-tagged SIRT3 or empty vector (EV). SIRT3 protein levels in whole cell lysates were detected by immunoblotting with anti-HA or anti-SIRT3 antibody. Actin serves as a loading control. (**b**) Osteoclast differentiation in BMMs infected with SIRT3 or EV. TRAP^+^MNCs (>3 nuclei) were counted. Scale bar, 500 μm. *P < 0.01. Data are represented as mean ± SD. (**c,d**) Expression of *Nfatc1* and NFATc1-induced genes *Atp6v0d2* during osteoclastogenesis. Infected BMMs were treated with RANKL for indicated days, and then subjected to qRT-PCR (**c**) or immunoblot analysis (**d**), respectively. Actin serves as a loading control. *P < 0.005 between the indicated groups. Data are represented as mean ± SD. (**e**) Bone resorption activity of WT or *Sirt3*^*−/−*^ osteoclasts. After differentiation of BMMs into osteoclasts, cells were seeded onto dentine discs and further incubated with RANKL for 3 days. The cells were removed from the dentine discs and stained with hematoxylin for visualization of pit formation. The area of resorption pits were measured with Image-Pro Plus 4.5 (Media Cybernetics). Data are represented as mean ± SD. Scale bar, 100 μm. n.s., not significant.

To further confirm the negative role of SIRT3 in RNAKL-induced osteoclast differentiation, BMMs were infected with retroviruses expressing SIRT3 or empty vector (Fig. 3a). The BMMs infected with SIRT3 but not the control (EV) had a significantly reduced capacity of forming TRAP-positive MNCs (Fig. 3b) along with a sizable reduction in the RANKL-dependent increase in *Nfatc1* and *Atp6v0d2* mRNA (Fig. 3c) and protein levels (Fig. 3d). These results confirmed that SIRT3 is a negative regulator of osteoclast differentiation.

Next, we examined whether ablation of SIRT3 affected their bone-resorbing activity. When we placed the same number of mature osteoclasts on dentin slices, there was no difference in the area of pit resorbed by WT and *Sirt3*−/− mature osteoclasts (Fig. 3e). Consistent with this, RANKL-induced actin ring formation in *Sirt3*−/− BMMs was also comparable with that of WT BMMs (Supplementary Fig. 3). Thus, these results suggested that SIRT3 was involved osteoclast differentiation but not in osteoclast function.

To gain an insight into the mechanism by which SIRT3 negatively regulated the RANKL-induced osteoclastogenesis, we examined the effects of SIRT3 deficiency on the activation of protein kinases downstream of RANKL or M-CSF by immunoblotting. Phosphorylation of ERK, p38, JNK, Akt, and IκB in response to RANKL occurred normally in osteoclast precursors of WT or *Sirt3*−/− BMMs (Supplementary Fig. 4a). Similarly, there was no significant difference in M-CSF-dependent signaling pathways, including MAP kinases and Akt, between WT and *Sirt3*−/− BMMs (Supplementary Fig. 4b). Neither the proliferation of osteoclast precursors nor cell surface expression of RANK and c-Fms during osteoclastogenesis were affected by SIRT3 deficiency (Supplementary Fig. 5a,b).

### Deletion of SIRT3 did not cause any defects in mitochondria of osteoclast precursors

Previous studies demonstrated that alteration in *Sirt3* expression regulates mitochondrial metabolism and production of ROS in skeletal muscle^24^,^27^. Since mitochondrial ROS produced by RANKL contributes to osteoclastogenesis^25^, we investigated whether ablation of *Sirt3* affected the production of mitochondrial ROS in BMMs by RANKL. Using a mitochondrial ROS-specific dye (MitoSOX), we observed no differences in the production of mitochondrial ROS between *Sirt3*−/− and WT BMMs (Supplementary Fig. 6a). In addition, cellular ROS levels in *Sirt3*−/− BMMs were comparable to those of control cells (Supplementary Fig. 6b). Since SIRT3, a mitochondrial deacetylase, is involved in the regulation of mitochondrial electron transport, we compared the activities of complex I and II (SDH) in isolated mitochondria from BMMs. There were no significant differences between WT and *Sirt3*−/− BMMs (Supplementary Fig. 7a,b). In addition, steady-state cellular ATP levels (Supplementary Fig. 7c) as well as mitochondrial content by MitoTracker staining (Supplementary Fig. 8) in Sirt3-depleted pre-osteoclasts were not significantly changed. These results suggest that SIRT3, unlike skeletal muscle or liver, was dispensable for the mitochondrial function in osteoclast precursors.

### PGC-1β-dependent induction of SIRT3 was observed during osteoclastogenesis

**Figure 4:**
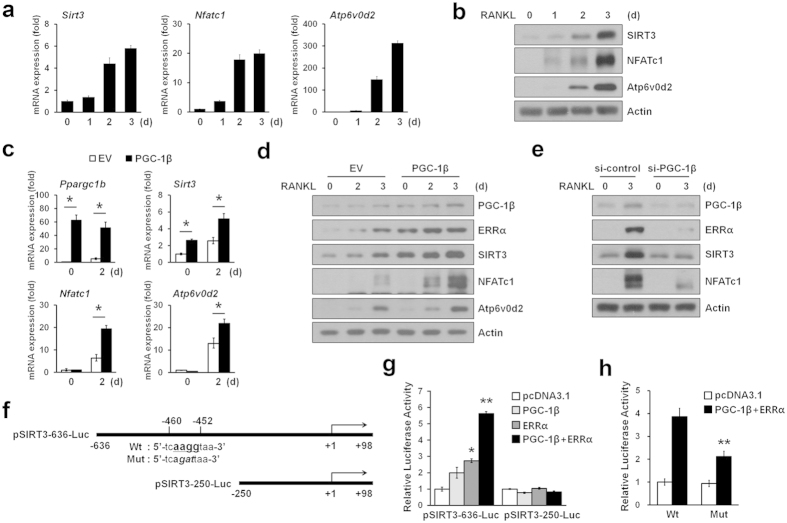
PGC-1β regulated the expression of SIRT3 during osteoclastogenesis. (**a**) BMMs were induced to differentiate into osteoclasts with RANKL for indicated days. Relative mRNA levels of *Sirt3*, *Nfatc1*, and NFATc1-induced genes *Atp6v0d2* were analyzed by qRT-PCR. (**b**) Immunoblot analysis of SIRT3, NFATc1, and Atp6v0d2 expression during osteoclast differentiation. Actin served as a loading control. (**c**) BMMs infected with retroviruses expressing PGC-1β or empty vector (EV) were stimulated with RANKL for 2 days. Total RNA was subjected to qRT-PCR analysis to assess *Ppargc1b*, *Sirt3*, *Nfatc1,* and *Atp6v0d2* mRNA levels. *P < 0.01 between the indicated groups. (**d**) As in (**c**), except that cell lysates were subjected to immunoblot analysis with the indicated antibodies. Immunoblot analysis showed that SIRT3 protein level was induced by PGC-1β. (**e**) BMMs were transiently transfected with either irrelevant siRNA (si-control) or PGC-1β siRNA and were stimulated with RANKL. Cell lysates were subjected to immunoblot analysis with the indicated antibodies. (**f**) Schematic representation of the mouse SIRT3 promoter-luciferase reporter constructs. pSIRT3-636-Luc containing a consensus ERRE site (Wt) and mutated ERRE site (Mut), respectively. (**g**) Expression plasmids for PGC-1β, ERRα or vector control were transfected into the HEK293T cells, along with the indicated pSIRT3-Luc constructs. The relative luciferase activity was corrected for Renilla luciferase activity and normalized to the vector control activity. (**h**) HEK293T cells were transiently co-transfected with a luciferase reporter plasmid containing a wild-type SIRT3 promoter construct pSIRT3-636-Luc or a mutant construct of the ERRE binding site together with the PGC-1β and ERRα expression plasmids. After 24 h incubation, cells were then assayed for relative luciferase activity. All values represent at least three independent transfections, each conducted in triplicate. Data represent means ± SD *P < 0.01, **P < 0.005.

We found that SIRT3 was only slightly expressed in osteoclast precursors, but was markedly induced in BMMs stimulated with RANKL (Fig. 4a,b). On the basis of the previous observations that PGC-1β regulates osteoclastogenesis^28^ and a PGC-1β homolog, PGC-1α and its coactivator ERRα induce transcription of SIRT3^29^, we examined the involvement of PGC-1β and ERRα in SIRT3 induction during osteoclastogenesis. As expected, the expression of PGC-1β and ERRα was enhanced during osteoclastogenesis for mRNA and protein levels (Supplementary Fig. 9a,b). We found that overexpression of PGC-1β along with RANKL stimulation significantly increased SIRT3 expression in both mRNA and protein levels (Fig. 4c,d). Consistently, PGC-1β knockdown led to the down-regulation of SIRT3 expression levels (Fig. 4e). However, neither overexpression of a constitutively active NFATc1 (ca-NFATc1) nor treatment of cyclosporin A (CsA), an inhibitor of calcineurin activity, changed SIRT3 levels (Supplementary Fig. 10a,b).

To determine how PGC-1β regulated the expression of SIRT3 at the transcriptional level, we cloned a 636-bp promoter of murine SIRT3 into a luciferase reporter pSIRT3-636-Luc (Fig. 4f). Indeed, there is a putative ERRα-binding DNA element^30^ in the 5′-flanking region (−460 to −452) of murine SIRT3 promoter. Transient transfection of either PGC-1β or ERRα activated the murine SIRT3 promoter by 2-fold or 2.8-fold, respectively. This activity was synergistically enhanced at about 6-fold by co-transfection of ERRα with PGC-1β (Fig. 4g). Furthermore, this induction was abolished when the putative ERRα-binding site of this promoter was deleted pSIRT3-250-Luc (Fig. 4g). In addition, ERRα and PGC-1β activation of the murine SIRT3 promoter was diminished upon ERRE mutation (Fig. 4h). These results suggest that both PGC-1β and ERRα were induced by RANKL and consequently increased SIRT3 at the transcriptional level.

### SIRT3 regulated AMPK stability

**Figure 5:**
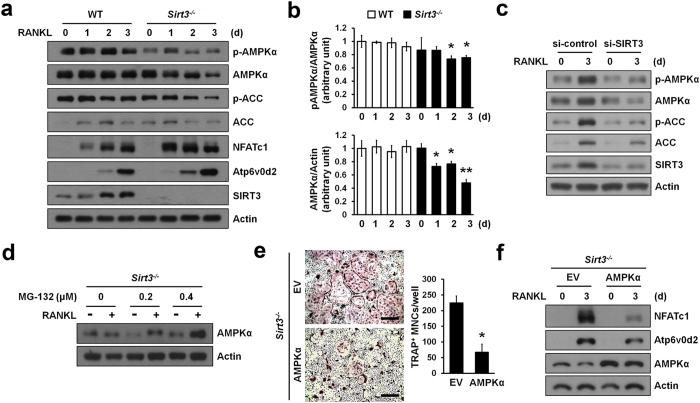
SIRT3 regulated AMPK stability. (**a**) BMMs from WT or *Sirt3*^*−/−*^ mice were stimulated with RANKL for the indicated time periods. Activation of AMPK or ACC and expression levels of NFATc1, Atp6v0d2, and SIRT3 during osteoclast differentiation. (**b**) The chemiluminescence signals for phospho-AMPK and AMPK were quantified and normalized based on the signals of AMPK and actin, respectively. Data represent means ± SD *P < 0.05, **P < 0.01. (**c**) RANKL stimulated BMMs transfected with si-control (scramble siRNA) or si-SIRT3 (siRNA against SIRT3) were immunoblotted with the indicated antibodies. (**d**) AMPK degradation in *Sirt3*^*−/−*^ osteoclasts is inhibited by proteasome inhibitor. BMMs from *Sirt3*^*−/−*^ mice were stimulated with RANKL in the absence or presence of MG-132 for 3 days. AMPK protein level was determined. Actin serves as a loading control. (**e**) Retroviral AMPKα introduction rescued osteoclast differentiation in *Sirt3*^*−/−*^ BMMs. *Sirt3*^*−/−*^ BMMs were transduced with empty or AMPKα retrovirus and then stimulated with RANKL. TRAP^+^MNCs (>5 nuclei) were counted. Scale bar, 500 μm. *P < 0.01. Data are represented as mean ± SD. (**f**) BMMs from *Sirt3*^*−/−*^ mice were transduced with retroviruses expressing AMPKα or EV and were treated with RANKL for 3 days. NFATc1 and Atp6v0d2 protein expression was determined by immunoblot analysis. Actin serves as a loading control.

On the basis of the previous observations that SIRT3 regulates the phosphorylation of AMPK in diet- or exercise-induced pathway^19^,^20^ and that AMPK deficiency is associated with increased osteoclastogenesis^31^,^32^, we examined the involvement of AMPK in linking osteoclastogenesis and SIRT3. Immunoblot analysis revealed that the phosphorylation of AMPKα at Thr172 was gradually decreased after stimulation with RANKL in *Sirt3*−/− BMMs (Fig. 5a,b). Since total protein levels of AMPKα, but not its mRNA levels (Supplementary Fig. 11) also changed by RANKL stimulation, it is probable that SIRT3 may have affected AMPKα at the post-translational levels. Consistent with this, acetyl-CoA carboxylase (ACC) that is phosphorylated by AMPK at Ser79^33^ was significantly suppressed in *Sirt3*−/− BMMs (Fig. 5a). To assess whether decreased expression of AMPK was a direct consequence of Sirt3 deficiency, we performed siRNA-mediated knockdown of *Sirt3* in BMMs. Knockdown of *Sirt3* resulted in marked reduction of total protein and phosphorylation levels of AMPKα and ACC (Fig. 5c). Protein stability of AMPKα was increased by treatment with a proteasome inhibitor MG-132 on *Sirt3*−/− BMMs (Fig. 5d). Finally, to clarify whether the reduced levels of AMPKα protein may be one of potential factors which are responsible for enhanced osteoclast differentiation in *Sirt3*−/− BMMs, we overexpressed AMPKα protein in *Sirt3*−/− BMMs using retroviral system. We found that osteoclastogenesis was significantly reduced in *Sirt3*−/− BMMs overexpressed with AMPKα compared with control (EV, Empty Vector) (Fig. 5e,f). These data suggested that SIRT3 served as a negative mediator of osteoclast differentiation through regulation of protein levels of AMPKα.

## Discussion

Mitochondrial ATP and ROS production as well as mitochondrial biogenesis play a key role in the regulation of osteoclast differentiation^25^,^26^,^28^. Though SIRT3 as a major mitochondrial deacetylase has been well documented to have a variety of mitochondrial functions such as energy production, ROS homeostasis, and mitochondrial biogenesis in several tissues^17^,^23^,^24^, the role of SIRT3 in osteoclast differentiation remains unexplored. In this study, we identified a new role for SIRT3 as negative regulator in osteoclast differentiation via regulation of AMPK activity. Our study revealed that SIRT3 deficiency significantly enhanced osteoclast differentiation, but did not affect osteoblast differentiation, suggesting that SIRT3 regulated bone homeostasis through modulating osteoclast differentiation. Consistent with the observed enhanced osteoclastogenesis, *Sirt3*−/− mice exhibited reduced bone mass due to an increased number of osteoclasts. Although our findings are not consistent with previous report^14^, it is most likely due to different experimental conditions, such as different mouse backgrounds used. We further demonstrated that SIRT3 was induced by coordination of PGC-1β and ERRα at the transcriptional level during osteoclast differentiation, and played a negative role on osteoclastogenesis through regulation of AMPK activity.

To date no report on the role of SIRT3 in osteoclast biology exists. With SIRT3 regulating mitochondrial functions^15^, we postulated a pro-osteoclastogenic role for SIRT3. However, SIRT3 deletion had no relevant effects on the mitochondrial function in osteoclast precursors. Specifically, we observed similar levels of cellular/mitochondrial ROS and of ATP in Sirt3-deficient osteoclast precursors compared with their wild-type counterparts, as well as similar levels of activity of the mitochondrial complex I and II (SDH). Whatever the underlying mechanism, these data imply that SIRT3 is dispensable for ATP or ROS homeostasis during osteoclast differentiation. Although many studies have demonstrated SIRT3 as a critical mediator in mitochondrial functions, some studies also reported normal ROS levels in basal state in liver, brain or cochlea^34^,^35^. In addition, Fernandez-Marcos *et al.*^36^ reported recently that the mitochondrial hyperacetylation induced by SIRT3 deletion in a tissue specific manner was not necessarily linked to mitochondrial dysfunction and did not recapitulate the metabolic abnormalities observed in the germline Sirt3 knock-out mice. Unlike the previous reports^14^,^17^, a few mitochondrial proteins in *Sirt3*−/− BMMs were shown to be hyperacetylated but most ones were similar with that in WT BMMs (Supplementary Fig. 12), implying that SIRT3 may have a unique role in osteoclast precursor cells unlike in other tissues such as liver and muscle. Also, this result may explain why mitochondrial functions of SIRT3 during osteoclastogenesis have no obvious difference between *Sirt3*−/− and WT BMMs.

Recently, Ishii *et al.*^28^ reported that PGC-1β coordinated with iron uptake to orchestrate mitochondrial biogenesis during osteoclast development. Moreover, PGC-1β functioned as a transcriptional coactivator for both PPARγ and ERRα to induce the expression of *c-fos* and mitochondrial genes^37^, thus linking osteoclast differentiation with osteoclast activation. It also has been reported that the expression of SIRT3 was stimulated by PGC1α, which serves as a crucial activator of mitochondrial biogenesis, and then, activated SIRT3 was required for PGC1α-mediated mitochondrial biogenesis in muscle cells and hepatocytes^29^. Similarly, we found that SIRT3 was induced at the transcriptional level during osteoclast differentiation depending on PGC-1β together with ERRα. Considering the role of SIRT3 in mitochondrial biogenesis^13^,^15^, it is probable that SIRT3 enhances osteoclast differentiation via the modulating mitochondrial biogenesis of osteoclasts. However, SIRT3 deletion in osteoclast precursors did not show any defects in mitochondrial biogenesis. SIRT3 even has a negative role in the terminal differentiation process through transcriptional suppression of genes such as *Ppargc1b* and *Nfatc1*.

Negative regulation of osteoclast differentiation plays an important role in controlling bone homeostasis and preventing development of bone-related diseases. Multiple regulatory mechanisms including a complicated network of transcriptional co-repressors^1^,^9^ and extracellular secreted molecules such as IFN-β and OPG have evolved to keep osteoclastogenesis in check. In this regard, we have provided functional evidence *in vitro* and *in vivo* that SIRT3 can serve as a new negative regulator of osteoclast differentiation. In addition, our present study also reveals a previously unrecognized role for PGC-1β in balancing osteoclastogenesis by inducing SIRT3 expression via PGC-1β/ERRα-dependent mechanism. Since excessive or inappropriate differentiation of osteoclasts can be deleterious to the organism, it is probable that PGC-1β induced its own inhibitor SIRT3 in an auto-regulatory feedback mechanism to keep osteoclastogenesis in check.

How might SIRT3 act as a negative regulator during osteoclast differentiation? As for one of the clues, our data showed that SIRT3 deletion reduced the activity of AMPKα, which is a cellular energy sensor^21^, by either protein stability or phosphorylation level. Consistent with this, the phosphorylation level of ACC, which is a target of AMPKα, was also lowered in *Sirt3*−/− cells compared to that of wild type cells during osteoclast differentiation. Recent studies have shown that the genetic or pharmacological inhibition of AMPKα enhanced osteoclastogenesis, indicating that AMP kinase acts as a negative regulator of RANKL in the differentiation of osteoclasts^31^,^32^. It is currently unclear how SIRT3 regulates the activity of AMPKα. A recent study showed that SIRT3 could activate AMPK through deacetylation and activation of LKB1^19^, which is a serine/threonine kinase and activator of AMPK. However, this seems unlikely because of different cellular localizations of SIRT3 and LKB1, one in mitochondria and the other in the cytoplasm, respectively. Furthermore, we also could not observe the co-localization of SIRT3 and LKB1 in osteoclast precursors (data not shown), indicating that yet unidentified factor(s) may be mechanically involved in SIRT3-mediated AMPKα activity.

In summary, this study demonstrates that SIRT3 negatively regulates osteoclastogenesis via an auto-regulatory feedback loop comprised of PGC-1β, ERRα and AMPK. In addition, the present work suggests that SIRT3 may be functioning as a molecular brake on the differentiation of osteoclasts. Further research into the molecular mechanism of SIRT3 action will be required to understand whether SIRT3 truly serves as a bridge between PGC-1β and AMPK. These efforts will not only provide us with a deeper understanding bone homeostasis but also might lead to new means for therapeutic intervention in bone diseases.

## Methods

### Mice

Sirt3 knockout mice were generated as described^17^. In this study, the *Sirt3*+/− mice^17^ were backcrossed with C57BL/6 (The Jackson Laboratory, Bar Harbor ME, USA) background for ten generations, and then interbred to generate complete deletion of exons 2−4 of Sirt3 (*Sirt3*−/− mice). Mice were genotyped by PCR analysis using the following primers: F1; 5′-gagatccatcagcttctgtg-3′, R1; 5′-ccctcaatcacaaatgtcgg-3′, F2; 5′-gggagcactctcatactcta-3′, R2; 5′-ttactgctgcctaacgttcc-3′. Primers F1 and R1 are located within intron 4 and amplify the wild-type allele (450 bp). Primers F2 and R2 are located within intron 1, and the combination of primers F2 and R1 amplify the deleted allele (486 bp). All experiments were approved by the Institutional Animal Care and Use Committee of Ewha Laboratory Animal Genomics Center, and were carried out in accordance with the approved guidelines.

### Cell culture and osteoclasts differentiation assay

Primary bone marrow-derived monocytes/macrophages (BMMs) were isolated from 6 to 8-week-old C57BL/6 male mice tibias and femurs as described^38^. In brief, bone marrow cells were cultured in α-minimum essential medium (α-MEM; HyClone, Logan UT, USA) supplemented with 10% fetal bovine serum (FBS; HyClone), 100 units/ml penicillin, and 100 μg/ml streptomycin for 12–18 h to remove adherent cells. The non-adherent cells were then harvested and cultured with 30 ng/ml M-CSF for 3 days. For osteoclast differentiation, isolated BMMs were stimulated with 100 ng/ml RANKL in the presence of M-CSF for 3–5 days. The cells were fixed with 3.7% formaldehyde in PBS and stained for tartrate-resistant acid phosphatase (TRAP) using the acid phosphatase, leukocyte (TRAP) kit (Sigma-Aldrich, St. Louis MO, USA) following the manufacturer’s instructions. TRAP-positive multinucleated cells (MNCs) were counted as osteoclast-like cells. Plat-E cells were maintained in Dulbecco’s modified Eagle’s medium (DMEM; HyClone) supplemented with 10% FBS, 100 units/ml penicillin/100 μg/ml streptomycin, 1 μg/ml puromycin (Sigma-Aldrich), and 10 μg/ml blasticidin (Invitrogen, Carlsbad CA, USA).

### Bone resorption assay

BMMs from WT and *Sirt3*−/− mice were incubated with 30 ng/ml M-CSF and 100 ng/ml RANKL for 3 days. Obtained pre-osteoclasts were seeded onto dentine discs (Immunodiagnostic Systems, Boldon, UK) and further incubated in the presence of M-CSF and RANKL for 3 days. The cells were removed from the dentine discs and the dentine discs were stained with hematoxylin. Pit areas were photographed under a light microscope and analyzed using Image-Pro Plus version 4.5 software (Media-Cybernetics, Rockville MD, USA).

### Microcomputed tomography (μCT) and histomorphometric analysis

To evaluate bone mass and architecture by microcomputed tomography, mouse tibiae were fixed and scanned using a Skyscan 1076 *in vivo* μCT scanner (Bruker Corporation, Karlsruhe, Germany). The structural parameters such as bone volume fraction (BV/TV), trabecular thickness (Tb.Th), trabecular separation (Tb.Sp), and trabecular number per unit length (Tb.N) were analyzed on three-dimensional images obtained by CT-An (Skyscan). For bone histomorphometric analyses, bones were fixed in 10% formaldehyde, decalcified in 0.5 M EDTA, pH 7.4, embedded in paraffin, and then cut into 4-μm sections. Hematoxylin and eosin (H&E) or TRAP staining was performed according to a standard protocol. The histomorphometric data were analyzed by Osteomeasure XP (OsteoMetrics Inc., Decatur GA, USA).

### siRNA transfection

Double-stranded, siRNAs targeting mouse SIRT3 and PGC-1β were synthesized from Genolution Pharmaceuticals Inc. (Seoul, Korea). The corresponding target mRNA sequences for the siRNAs were as follows: si-SIRT3, GCGTTGTGAAACCCGACAT; si-PGC-1β, CCTTCCAATATGTTTACGTT; scrambled nontargeting siRNA, ACGTGACACGTTCGGAGAA, as a negative control. BMMs were transfected with the gene-specific siRNA at a concentration of 10 nM using Lipofectamine^TM^ RNAiMAX (Invitrogen) according to the manufacturer’s protocol.

### Retroviral infection

The pMX-puro vector and Platinum-E (Plat-E) packaging cells were previously described^39^. Plat-E cells were transfected with pMX-puro empty vector, pMX-SIRT3-HA, pMX-PGC-1β, or pMX-AMPKα retroviral expression vectors by using polyethylenimine (PEI; Sigma-Aldrich) reagent. After 48 hours, the supernatants were collected and filtered through 0.45 μm filters (BD Biosciences, Franklin Lakes NJ, USA). BMMs were transduced with reteoviral particles supplemented with polybrene (10 μg/ml; Sigma-Aldrich) for 12 hours. After transduction, cells were cultured in the presence of M-CSF and 2 μg/ml puromycin to select for infected cells for 2 days. Puromycin-resistant cells were used in all the experiments described.

### Semi quantitative real time PCR

Total RNA was extracted from BMMs by TRIzol reagent (Invitrogen) and first-strand cDNA was synthesized with oligo (dT) primers and M-MLV reverse transcriptase (SolGent, Seoul, Korea). The relative mRNA levels were evaluated by quantitative RT-PCR (qRT-PCR) using SYBR Green Master kit (Kapa Biosystems, Woburn MA, USA). Gene specific primer sequences are provided in Supplementary Methods.

### Western blot analysis

BMMs were lysed and processed by standard methods detailed in Supplementary Methods.

### Transfection and luciferase reporter assay

The mSIRT3 promoter was amplified using mouse genomic DNA and cloned at the *Kpn* I and *Xho* I sites of the pGL3-Basic vector (Promega, Fitchburg WI, USA). To amplify different promoter regions, the corresponding forward primers 5′-ACATTGGGTACCGACTCTGCTGTAAGAAGGCCGGCA-3′ pSIRT3-636-Luc, 5′- ATTCTCGGTACCCGCCTACTCAAGGAGGTCGAGAGC-3′ pSIRT3-250-Luc were used with the identical reverse primer 5′-ATTCAGCTCGAGCCCACAGTCTGAGCGCGGCCAATG-3′ (+98 bp). Site-direct mutagenesis was performed using Pfu DNA polymerase (SolGent, Seoul, Korea). HEK293T cells were cotransfected with PGC-1β plasmid, ERRα plasmid and luciferase reporter construct. The mass of transfected plasmids was balanced with empty vector (pcDNA3.1). Luciferase activity was measured by the dual luciferase assay system (Promega) according to the manufacturer’s protocol and normalized to the activity of the control (pRenilla). The data were obtained from three independent transfections and presented as the -fold induction in luciferase activity (mean ± SD) relative to the control.

### Statistical analysis

Data were expressed as mean ± SD from at least three independent experiments. Statistical analyses were performed using the two-tailed Student’s *t* test to analyze differences among groups. *P* < 0.05 was considered statistically significant.

## Additional Information

**How to cite this article**: Huh, J.-E. *et al.* Sirtuin 3 (SIRT3) maintains bone homeostasis by regulating AMPK-PGC-1β axis in mice. *Sci. Rep.* **6**, 22511; doi: 10.1038/srep22511 (2016).

## Supplementary Material

Supplementary Information
