# Supplementary material for: Sirtuin 3 (SIRT3) maintains bone homeostasis by regulating AMPK-PGC-1β axis in mice
Source: Sci Rep. 2016 Mar 1;6:22511. doi: 10.1038/srep22511 (PMC4772385; doi:10.1038/srep22511)
Supplement: Supplementary Information [file srep22511-s1.pdf]

Supplementary Information

Sirtuin 3 (SIRT3) maintains bone homeostasis by regulating AMPK-PGC-1β axis in mice

Jeong-Eun Huh, Ji Hye Shin, Eun Sun Jang, So Jeong Park, Doo Ri Park, Ryeojin Ko, Dong-Hyun Seo, Han-Sung Kim, Seoung Hoon Lee, Yongwon Choi, Hyun Seok Kim, and Soo Young Lee

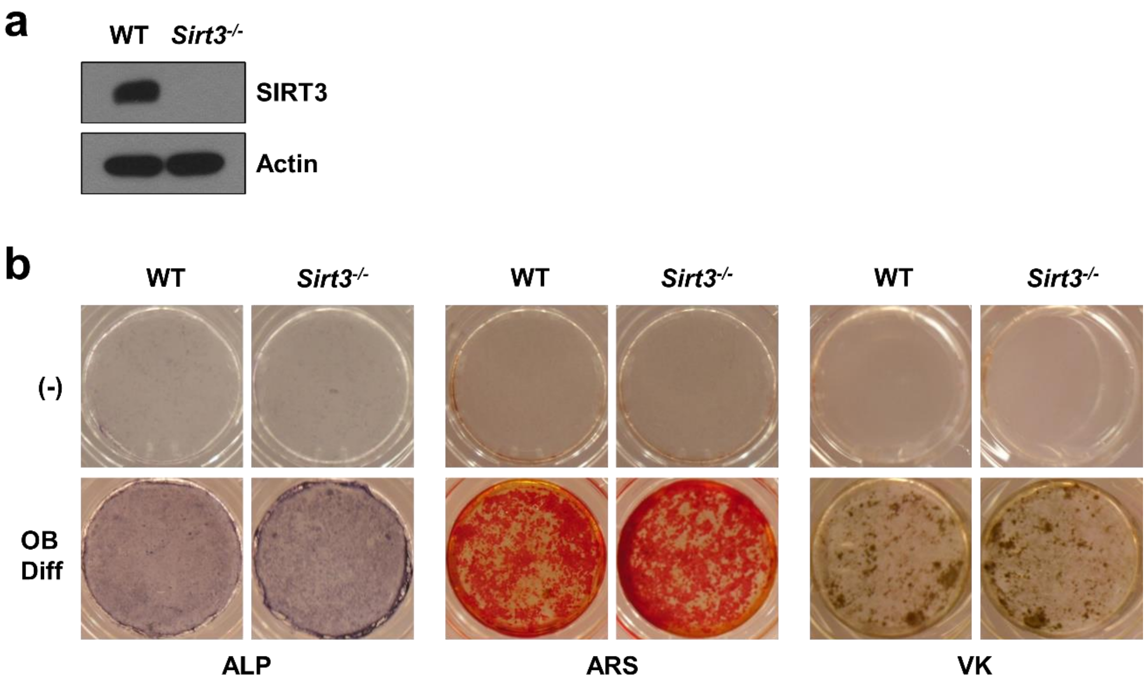

Supplementary Figure 1. SIRT3 had no effects during osteoblast differentiation.

(**a**) Immunoblot analysis of SIRT3 protein in BMSCs purified from the bone marrow of WT and *Sirt3*$^{-/-}$ mice. (**b**) BMSCs from WT or *Sirt3*$^{-/-}$ mice were cultured with osteogenic medium for 2 weeks. Alkaline phosphatase activity and matrix mineralization were visualized by ALP (*Left*), Alizarin red S (*Center*), or von Kossa (*Right*) staining.

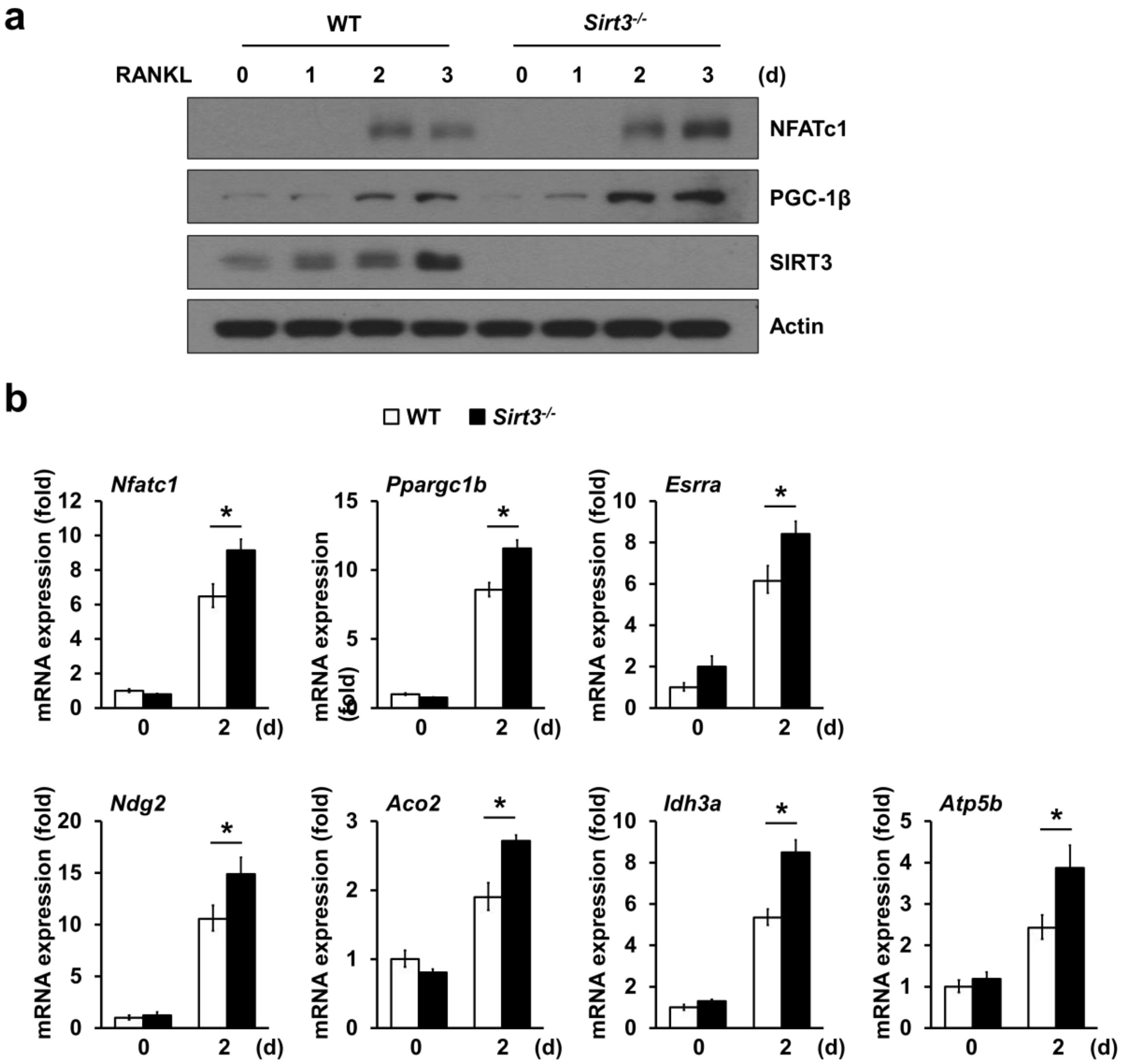

Supplementary Figure 2. SIRT3 deficiency enhanced PGC-1β and its target genes expression during osteoclastogenesis.

(**a**) BMMs from WT or *Sirt3*$^{-/-}$ mice were stimulated with RANKL for the indicated days. The cell lysates were subjected to immunoblot analysis using antibodies against NFATc1, PGC-1β, and SIRT3. Actin serves as a loading control. (**b**) BMMs from WT or *Sirt3*$^{-/-}$ mice were incubated with RANKL for 2 days. Total RNA was extracted and used in qRT-PCR to quantify the mRNA levels of *Ppargc1b* and its downstream targets of mitochondrial genes. *$P < 0.01$ between the indicated groups. Data are represented as mean ± SD. Ndg2; Nur77 downstream gene 2, Aco2; aconitase 2, Idh3a; isocitrate dehydrogenase 3, Atp5b; ATP synthase 5b

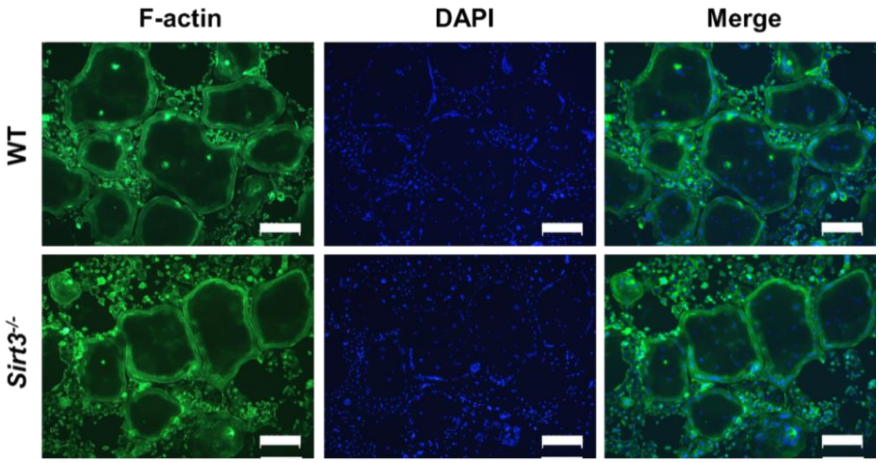

Supplementary Figure 3. RANKL-induced actin ring formation in WT or *Sirt3*$^{-/-}$ cells was comparable.

F-actin ring forming in WT or *Sirt3*$^{-/-}$ osteoclasts. WT or *Sirt3*$^{-/-}$ osteoclasts grown on glass coverslips and stained with phalloidin (green). DAPI (blue) shown nuclei. Scale bar, 200 μm.

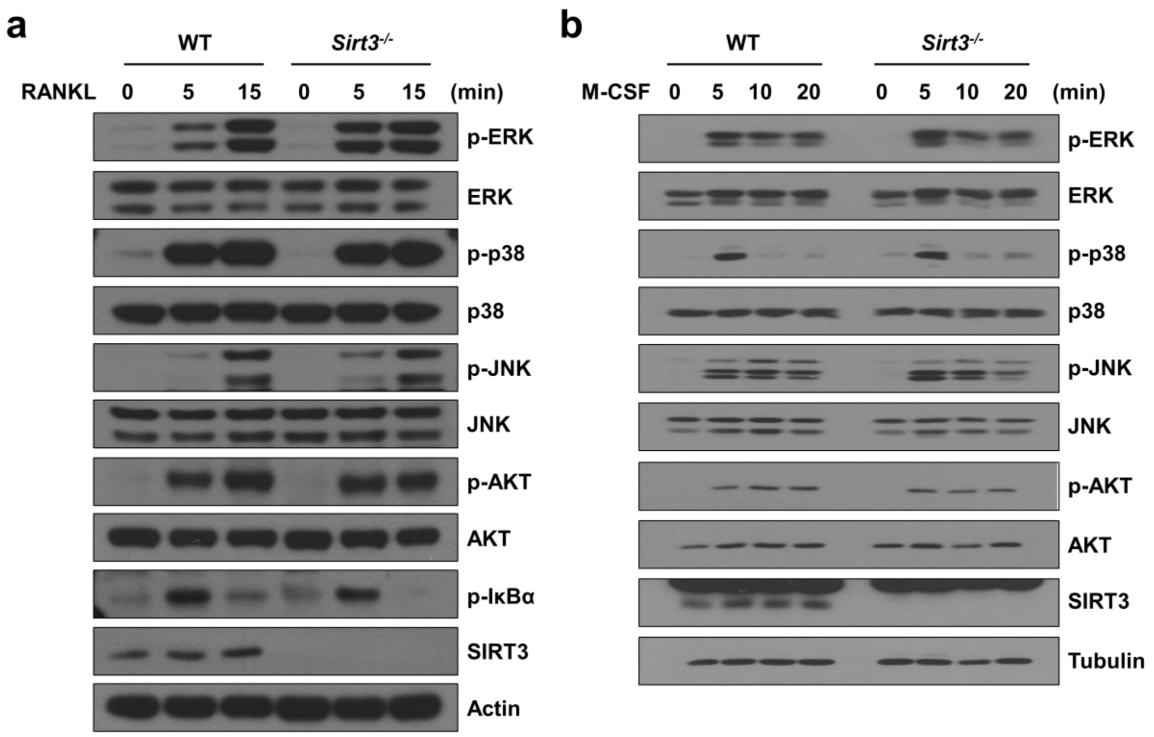

Supplementary Figure 4. RANKL or M-CSF-dependent signaling pathways between WT and *Sirt3*$^{-/-}$ cells were comparable.

(**a**) BMMs from WT or *Sirt3*$^{-/-}$ mice were stimulated with RANKL for the indicated times. The cell lysates were subjected to immunoblot analysis using antibodies against phospho-ERK, phospho-p38, phospho-JNK, phosphor-AKT, and phospho-IκBα. Immunoblots were stripped and then reprobed with total ERK, p38, JNK, and AKT. Actin serves as a loading control. (**b**) BMMs from WT or *Sirt3*$^{-/-}$ mice were exposed to M-CSF for the indicated times. The cell lysates were subjected to immunoblot analysis as described for panel A. Tubulin served as a loading control.

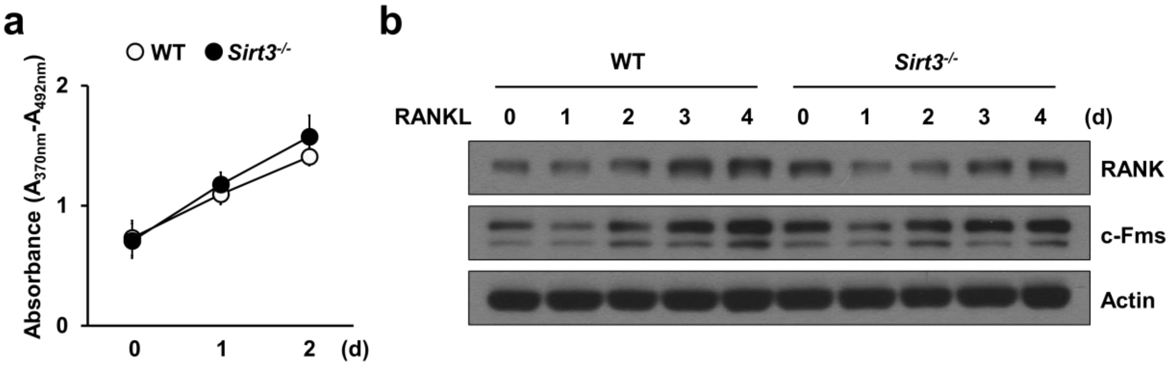

Supplementary Figure 5. Proliferation and expression of RANK or c-Fms between WT and *Sirt3*$^{-/-}$ cells were not altered.

(**a**) BMMs from WT or *Sirt3*$^{-/-}$ mice were cultured in the presence of M-CSF and RANKL for the indicated times. Proliferation was assessed by the absorbance of incorporated BrdU in WT and *Sirt3*$^{-/-}$ BMMs. (**b**) BMMs from WT or *Sirt3*$^{-/-}$ mice were stimulated with RANKL for the indicated days. The cell lysates were subjected to immunoblot analysis of RANK and c-Fms. Actin serves as a loading control.

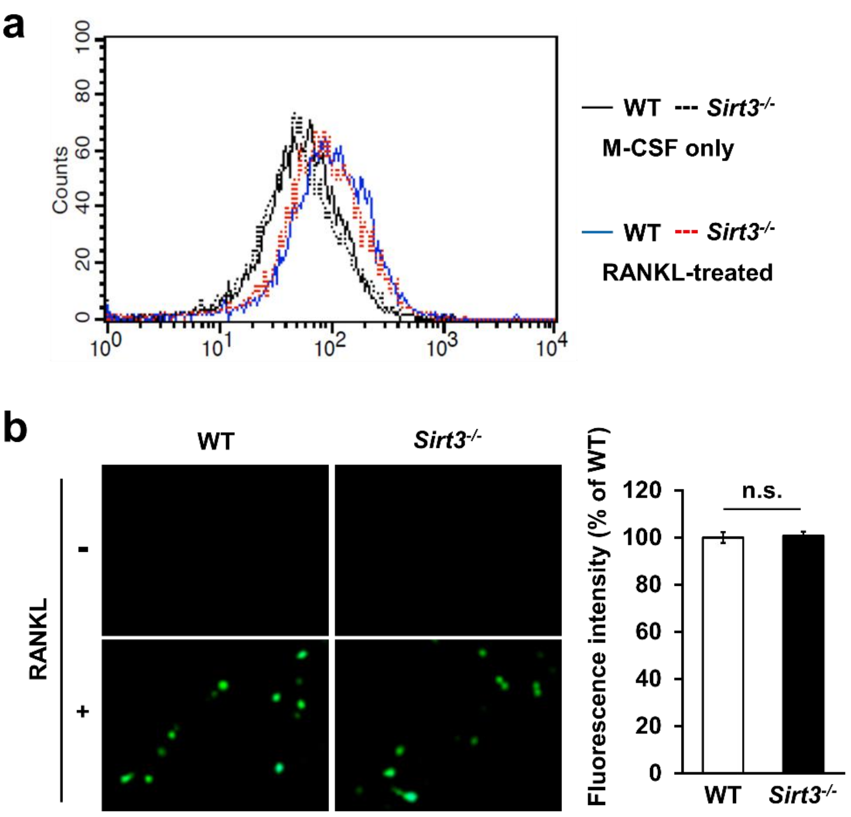

Supplementary Figure 6. RANKL-induced ROS production in WT and *Sirt3*$^{-/-}$ cells was comparable.

(**a**) Comparison of RANKL-mediated mitochondrial ROS production. BMMs from WT or *Sirt3*$^{-/-}$ mice were treated with RANKL for 24 h and then stained with MitoSOX for 10 min and analyzed by flow cytometry. Histograms show the results of one representative experiment out of three independent experiments. (**b**) RANKL-induced intracellular ROS in WT or *Sirt3*$^{-/-}$ BMMs. Cells were stimulated with 200 ng/ml RANKL for 10 min. The intracellular ROS was examined by a fluorescence microscope (*Left*) and a fluorescence spectrophotometer using DCF-DA (*Right*). Scale bar, 100 μm. Data are represented as mean ± SD. n.s., not significant.

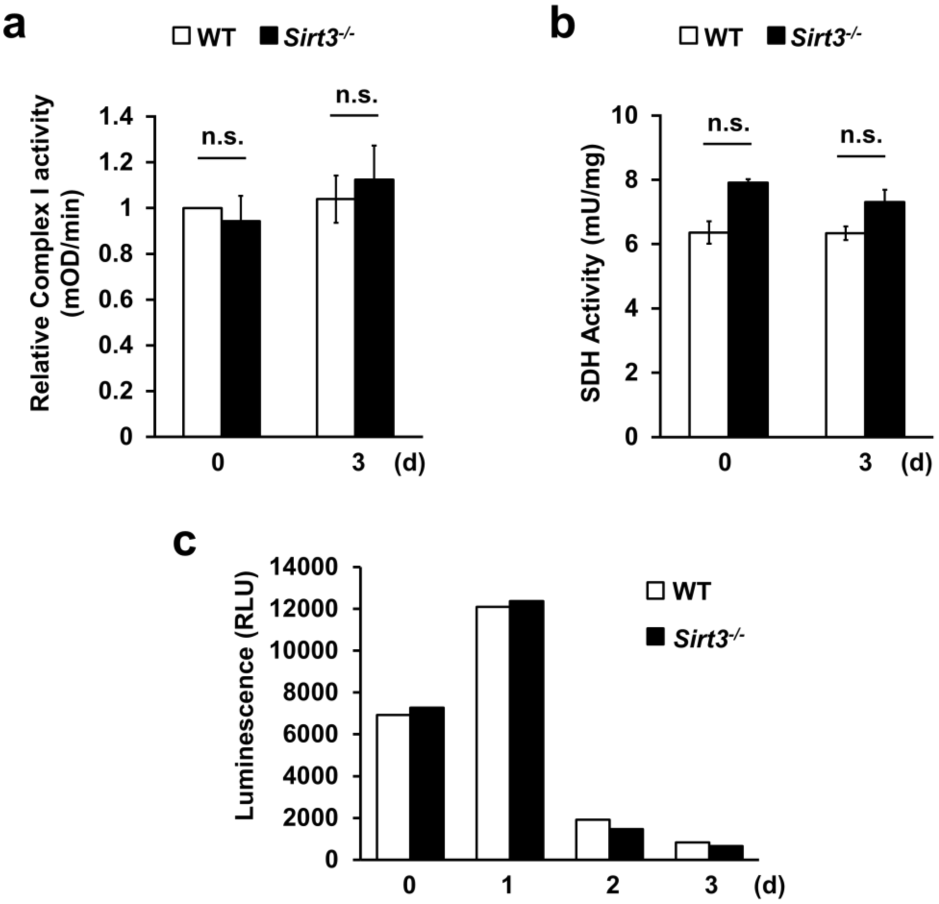

Supplementary Figure 7. Mitochondrial complex I or II activity and intracellular ATP levels between WT and *Sirt3$^{-/-}$* cells were comparable.

(**a,b**) Mitochondrial complex I and II activity measurement in BMMs from WT or *Sirt3$^{-/-}$* mice. BMMs were incubated with M-CSF with or without RANKL for the indicated days and fractionated into cytoplasm and mitochondria. Mitochondrial fractions were subjected to activity assay for (**a**) complex I and (**b**) complex II (SDH). Both enzyme activity was normalized using mitochondrial content. Data are represented as mean ± SD. n.s., not significant. (**c**) BMMs from WT or *Sirt3$^{-/-}$* mice were cultured with RANKL and cell lysates were collected on the indicated days and then measured intracellular ATP levels. Data show one experiment representative of three independent experiments.

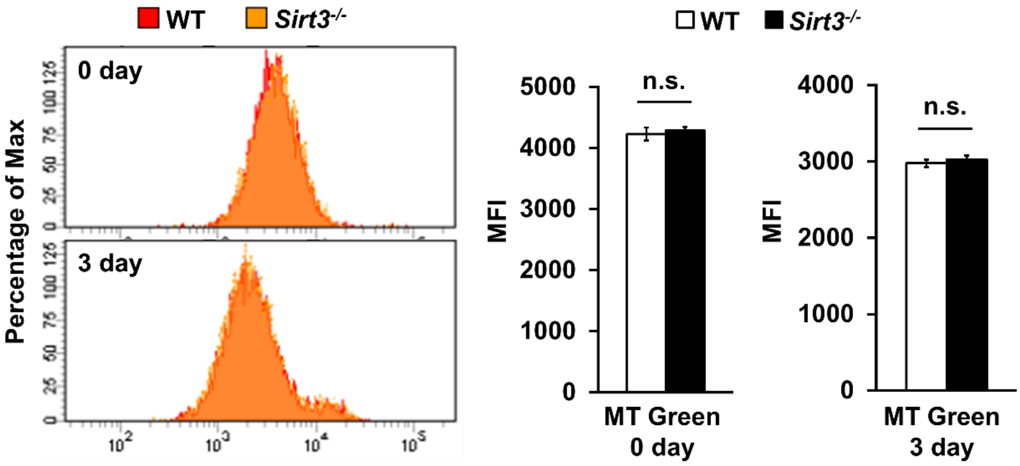

Supplementary Figure 8. Mitochondrial contents between WT and *Sirt3$^{-/-}$* cells were comparable.

BMMs from WT or *Sirt3$^{-/-}$* mice were cultured in the presence of M-CSF and RANKL for 3 days. Cells were washed with PBS and then incubated with 100 nM MitoTracker (MT) Green FM dyes at 37 °C for 15 min. The cells were washed 3 times in PBS and subjected to flow cytometric analysis. Representative histogram (*Left*) and mean fluorescence intensity (MFI, *Right*) for each sample are shown. Data are represented as mean ± SD. n.s., not significant.

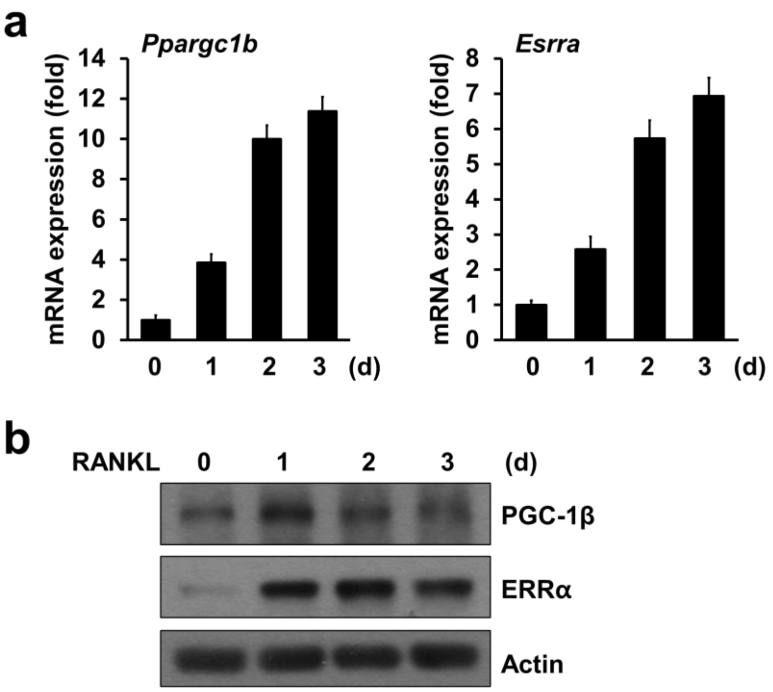

Supplementary Figure 9. Expression of PGC-1β and ERRα was increased by RANKL during osteoclastogenesis.

(**a**) BMMs were incubated with RANKL for indicated days. Total RNA was extracted from the cells and used in qRT-PCR to quantify the mRNA levels of *Ppargc1b* and *Esrra* genes. (**b**) Immunoblot analysis of PGC-1β and ERRα expression during osteoclast differentiation. Actin served as a loading control.

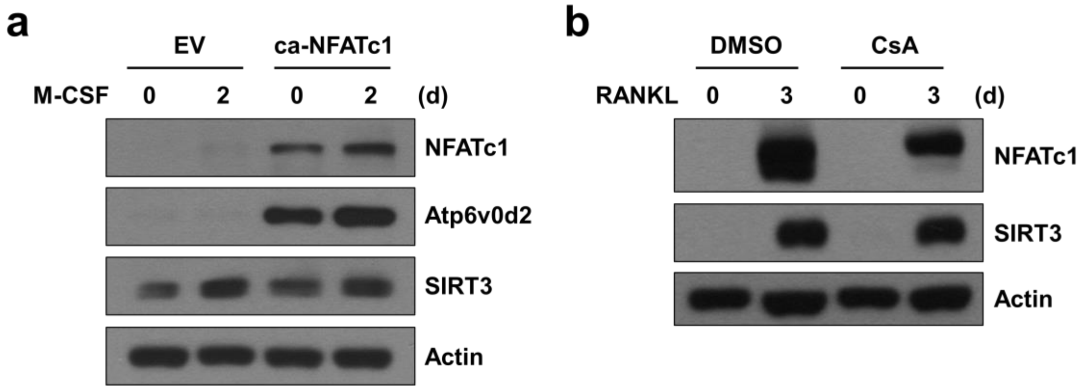

Supplementary Figure 10. NFATc1 did not induce the expression of SIRT3.

(**a**) Effect of NFATc1 on the expression of SIRT3. BMMs infected with retroviruses expressing constitutively active form of NFATc1 (ca-NFATc1) or empty vector (EV) were cultured with M-CSF in the absence of RANKL for 2 days, and then immunoblot analysis were performed. (**b**) BMMs were treated with DMSO or 1 μM cyclosporine A (CsA) in the presence of RANKL for 3 days. The cell lysates were subjected to immunoblot analysis of NFATc1 and SIRT3. Actin serves as a loading control.

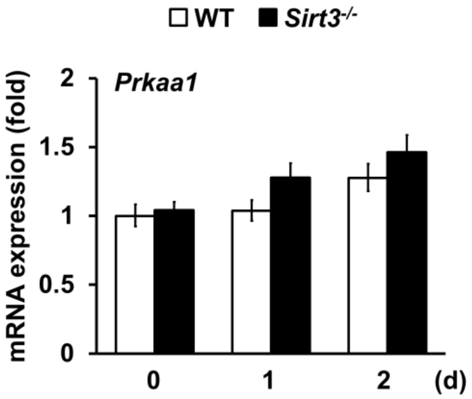

Supplementary Figure 11. RANKL-induced *Prkaa1* mRNA levels in WT and *Sirt3*$^{-/-}$ cells were comparable.

BMMs from WT or *Sirt3*$^{-/-}$ mice were treated with RANKL for indicated days. The expression of *Prkaa1* mRNA encoding AMPKα1 protein was measured using qRT-PCR.

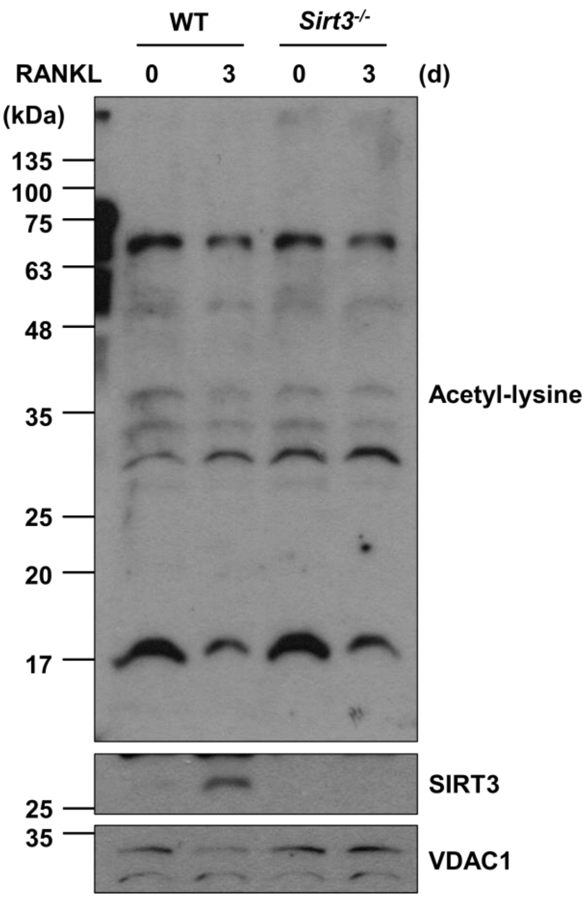

Supplementary Figure 12. Representative immunoblot of mitochondrial protein acetylation in WT and *Sirt3*$^{-/-}$ cells.

BMMs from WT or *Sirt3*$^{-/-}$ mice were incubated with RANKL for 3 days and mitochondrial proteins were extracted. Mitochondrial lysates were subjected to immunoblot analysis using an anti-Acetyl-lysine antibody. VDAC1 serves as a loading control.

Supplementary Methods

Osteoblast differentiation.

Primary bone marrow-derived mesenchymal stromal cells (BMSCs) were isolated from 3 to 4-week-old C57BL/6 mice as described previously[1]. In brief, mice tibiae and femurs were cut into chips and digested with 0.1 % collagenase II (Sigma-Aldrich), then the bone chips were placed in cell culture dishes in α-MEM containing 10 % FBS, 100 units/ml penicillin, and 100 μg/ml streptomycin. After 5 days, adherent cells were harvested by trypsinization and further cultured. For osteogenic differentiation, isolated BMSCs were cultured in osteogenic medium containing 10 mM β-glycerol phosphate (Sigma-Aldrich), 50 μg/ml ascorbate-2-phosphate (Sigma-Aldrich), 10^{-7} M dexamethasone (Sigma-Aldrich) and 25 ng/ml human recombinant bone morphogenetic protein 2 (BMP-2, R&D Systems, Minneapolis MN, USA) for 2 weeks. Alkaline phosphatase (ALP) activity was detected by BCIP/NBT color development substrate (Promega) according to the manufacturer's instructions. The degree of extracellular matrix calcification was estimated using Alizarin red S (2 % ARS, pH 4; Alphachem, Middlesex, UK) and von Kossa (1 % silver nitrate solution; Sigma-Aldrich) staining.

Semi quantitative real time PCR.

BMMs were cultured with 30 ng/ml M-CSF with or without 100 ng/ml RANKL for the indicated time periods. Total RNA was extracted using TRIzol reagent (Invitrogen) according to the manufacturer's instructions. After denaturation of total RNA at 70 ℃ for 10 min, first-strand cDNA was synthesized with oligo (dT) primers and M-MLV reverse transcriptase (SolGent, Seoul, Korea). The relative mRNA levels were evaluated by quantitative RT-PCR (qRT-PCR) using SYBR Green Master kit (Kapa Biosystems, Woburn MA, USA). Reactions were performed in triplicate on ABI PRISM 7300 unit (Applied Biosystems, Carlsbad CA,

USA). The relative expression levels were calculated using the comparative C_T method ($\Delta\Delta C_T$) and values were normalized to actin levels as an internal control gene. Melting curve analysis was included to assure that only one PCR product was formed. The primer sequences are as follows: *Sirt3*, 5′-GCTGCTTCTGCGGCTCTATAC-3′ and 5′-GAAGGACCTTCGACAGACCGT-3′; *Nfatc1*, 5′-CCAGAAAATAACATGCGAGCC-3′ and 5′-GTGGGATGTGAACTCGGAAG-3′; *Atp6v0d2*, 5′-CAGAGATGGAAGCTGTCAACATTG-3′ and 5′-TGCCAAATGAGTTCAGAGTG-3′; *Oscar*, 5′-CGTTGAGCTGGCTGAGTTCT-3′ and 5′-TCTGGGGAGCTGATCCGTTA-3′; *Ppargc1b*, 5′-CTCCAGGCAGGTTCAACCC-3′ and 5′-GGGCCAGAAGTTCCCTTAGG-3′; *Esrra*, 5′-TTCGGCGACTGCAAGCTC-3′ and 5′-CACAGCCTCAGCATCTTCAATG-3′, and *Prkaa1*, 5′-CTACCTAGCAACCAGCCCAC-3′ and 5′-GCCATTTTGCCTTCCGTACA-3′.

Western blot analysis.

BMMs were cultured with 30 ng/ml M-CSF with or without100 ng/ml RANKL for the indicated time periods. Cells were then lysed in cell lysis buffer (50 mM Tris-HCl (pH 7.4), 150 mM NaCl, 0.5 % sodium deoxycholate, 1 % Nonidet P-40, 1 mM EDTA, 10 % glycerol) supplemented with 1 mM phenylmethylsulfonyl fluoride, 1 μg/ml of leupeptin, aprotinin, and pepstatin A, 1 mM sodium orthovanadate and 1 mM NaF for 20 min on ice. The lysates were centrifuged at 14,000 x g for 20 min at 4 °C, and then total protein concentration was determined by the Bradford assay (Bio-Rad, Hercules CA, USA). Equal amounts of proteins were mixed with SDS sample buffer, denatured for 5 min at 95 °C, and then resolved by SDS-PAGE. The resolved proteins were transferred to a PVDF membrane (Millipore, Billerica MA, USA) by electroblotting and subjected to immunoblotting with the indicated antibodies. Proteins were detected using an ECL detection kit (Amersham Biosciences,

Piscataway NJ, USA). Primary antibodies used in this study included anti-hemagglutinin (HA), anti-SIRT3, anti-ERRα, anti-AMPKα, anti-phospho-AMPKα (Thr172), anti-ACC, anti-phospho-ACC (Ser79), anti-ERK, anti-phospho-ERK (T202/Y204), anti-p38, anti-phospho-p38 (T180/Y182), anti-JNK, anti-phospho-JNK (T183/Y185), anti-AKT, anti-phospho-AKT (S473), anti-phospho-IκB (Ser32/36) (Cell Signaling Technology, Danvers MA, USA), anti-NFATc1, anti-PGC-1β, anti-c-Fms, anti-VDAC1, anti-β-actin, anti-α-tubulin (Santa Cruz Biotechnology, Santa Cruz CA, USA), anti-Acetyl-lysine (Millipore), and anti-RANK (R&D Systems). Anti-Atp6v0d2 antibody was kindly provided by Y. Choi (University of Pennsylvania).

Actin-ring staining.

After osteoclast differentiation, the cells were fixed in 4 % paraformaldehyde in PBS and further permeabilized with 0.1 % Triton X-100 for 20 min. Cells were incubated with Alexa Fluor 488-phalloidin (Invitrogen) for 20 min. After being washed with PBS, cells were stained with 4',6-diamidino-2-phenylindole (DAPI) and were photographed under a fluorescence microscope.

Evaluation of cell proliferation.

To confirm proliferative effects in WT and *Sirt3*$^{-/-}$ BMMs, BrdU cell proliferation kit (Roche Applied Science, Penzberg, Germany) was used. Cells were cultured with 30 ng/ml M-CSF with or without 100 ng/ml RANKL for the indicated time periods. Each sample was assayed in triplicate. After that, BrdU was added and the cells were reincubated for 4 h. After removing the culture medium, cells were fixed and the DNA denatured. Then, peroxidase-conjugated anti-BrdU was added to bind to the BrdU. The immune complexes were detected by the 3,3’,5,5’ tetramethylbenzidine substrate reaction, and the resultant color was read at

370 nm in a microplate spectrophotometer. The absorbance values correlated directly to the amount of DNA synthesis and thereby to the number of proliferating cells in culture.

Mitochondrial ROS measurement.

ROS levels were determined by flow cytometry using the MitoSOX Red mitochondrial superoxide indicator (Thermo Fisher, Waltham MA, USA). Once in the mitochondria, MitoSOX red reagent is oxidized by superoxide and exhibits red fluorescence. BMMs were stimulated with RANKL for 24 h and then stained with 5 μM MitoSOX reagent for 10 min at 37 °C and then washed twice with Hank's balanced salt solution (HBSS). Detached cells were washed once with PBS and resuspended in PBS containing 2 % FBS and 0.7 mM EDTA (disodium salt), and analyzed using a FACSCalibur (BD Biosciences, Heidelberg, Germany).

Detection of intracellular ROS.

Intracellular production of ROS was assayed as described[2]. In brief, 10 min after stimulation with 100 ng/ml RANKL, dishes of confluent cells were washed with α-MEM lacking phenol red and then incubated in the dark for 5 min in Krebs-Ringer solution containing 10 μM DCF-DA. Culture dishes were transferred to a Zeiss Axiovert 135 inverted microscope (Carl Zeiss, Oberkochen, Germany), and DCF (2′,7′-dichlorofluorescein) fluorescence was measured with an excitation wavelength of 488 nm and emission at 515-540 nm. After collection of the fluorescence image, the mean relative fluorescence intensity for each group of cells was then measured by Carl Zeiss vision system (KS400, version 3.0).

Mitochondria purification and Complex I/II activity assay.

Mitochondria from WT or *Sirt3*$^{-/-}$ BMMs were isolated using Qproteome Mitochondria Isolation Kit (QIAGEN, Venlo, Netherlands) following manufacture's protocol. Isolated mitochondrial fraction was subjected to enzymatic analysis of mitochondrial complex I and II activity using Mitochondrial Complex I Activity Assay Kit (AAMT001-1KITCN; MERK,

Whitehouse Station NJ, USA) and Succinate dehydrogenase activity assay kit (K660-100; Biovision, Milpitas CA, USA) following manufacture's protocol.

ATP measurement.

BMMs isolated from WT or *Sirt3*$^{-/-}$ mice were stimulated with 100 ng/ml RANKL for the indicated time periods, and lysed in ice-cold ATP buffer (20 mM Tris (pH 7.5), 0.5 % Nonidet P-40, 25 mM NaCl, 2.5 mM EDTA) for 5 min. Lysates were then centrifuged and the supernatant determined for protein concentration. ATP was measured by using the ATP Assay system with bioluminescence detection kit (Promega) according to the manufacturer's protocol. The luminescence was measured by a luminometer (Berthold Technologies), and the results were compared to the standards. The relative ATP level was then calculated by dividing the luminescence by the total protein concentration. Each ATP sample was measured in triplicate.

Measurement of mitochondria content.

BMMs from WT or *Sirt3*$^{-/-}$ mice were incubated in the presence of M-CSF and RANKL (50 ng/ml). After 3 days of incubation, cells were washed with PBS and incubated at 37 °C for 15 min with 100 nM MitoTracker Green FM dyes (Molecular Probes, Eugene OR, USA). Samples were washed 3 times in PBS and collected to 5 ml round-bottom tube and subjected to flow cytometric analysis on a FACSCalibur apparatus (Becton Dickinson, San Jose CA, USA).

Supplementary References

1. Zhu, H. *et al.* A protocol for isolation and culture of mesenchymal stem cells from mouse compact bone. *Nat Protoc* **5,** 550-560 (2010).

2. Lee, N. K.*et al.* A crucial role for reactive oxygen species in RANKL-induced osteoclast differentiation. *Blood* **106,** 852-859 (2005).
